# Characteristic strategy of assimilation of various saccharides by *Clostridium cellulovorans*

**DOI:** 10.1186/s13568-016-0237-5

**Published:** 2016-09-01

**Authors:** Takako Inamori, Shunsuke Aburaya, Hironobu Morisaka, Kouichi Kuroda, Mitsuyoshi Ueda

**Affiliations:** 1 Division of Applied Life Sciences, Graduate School of Agriculture, Kyoto University, Sakyo-ku, Kyoto, Japan; 2 Research Fellow of Japan Society for the Promotion of Science, Sakyo-ku, Kyoto, Japan; 3 Kyoto Integrated Science and Technology Bio-Analysis Center, Shimogyo-ku, Kyoto, Japan

**Keywords:** *Clostridium cellulovorans*, Polysaccharide assimilation, Cellulose, Hemicellulose

## Abstract

*Clostridium cellulovorans* can effectively assimilate not only cellulose but also hemicellulose by producing cellulosomal and non-cellulosomal enzymes. However, little is known about how *C. cellulovorans* assimilates various saccharides in media containing polysaccharides and oligosaccharides. In this research, we investigated the property of saccharide incorporation and assimilation by *C. cellulovorans*. Faster growth in media containing xylan and cellulose was achieved by switching polysaccharides, in which xylan was first assimilated, followed by cellulose. Furthermore, the presence of polysaccharides that can be easily degraded might increase the assimilation rate of lignocellulose by promoting growth. These properties of *C. cellulovorans* could be suitable for the effective utilization of lignocellulosic biomass.

**Electronic supplementary material:**

The online version of this article (doi:10.1186/s13568-016-0237-5) contains supplementary material, which is available to authorized users.

## Introduction

Lignocellulosic biomass is an attractive source because it is renewable, abundant, and does not compete with food. Lignocellulosic biomass is composed mainly of cellulose (40–50 %), hemicellulose and pectin (25–30 %), and lignin (15–20 %) (Gray et al. 2006). Cellulose is a polysaccharides comprised of a linear chain of glucose monomers firmly held together by hydrogen bonding. Its strong crystalline structure is difficult to efficiently degrade (Brethauer and Studer 2015). Hemicellulose consists of a branched polymer of various monosaccharides such as xylose, mannose, galactose, and arabinose. Hydrogen-bonded cellulose fibers are cross-linked by hemicellulose and pectin, and lignin increases its mechanical strength. These rigid and complex structures have made it difficult to degrade lignocellulosic biomass. Utilization of non-cellulosic polysaccharides (hemicellulose and pectin) affects the cost to produce useful materials. Therefore, utilization of non-cellulosic polysaccharides, especially xylose (the major component of hemicellulose), is important for optimal utilization of resources (Tamaru et al. 2010). Furthermore, improving the efficiency of simultaneous degradation of various polysaccharides through the degradation of non-cellulosic polysaccharides is necessary for promoting the utilization of lignocellulosic biomass.

Clostridia such as *Clostridium thermocellum, C. cellulolyticum*, and *C. cellulovorans* have received attention for their utilization of lignocellulosic biomass. These species have the ability to efficiently degrade biomass components by the assembly of a cellulolytic multi-enzyme complex called the cellulosome (Felix and Ljungdahl 1993) and by secreting different types of carbohydrases, and non-cellulosomal enzymes (Doi and Kosugi 2004). Among those clostridia, we focused on *C. cellulovorans*, which is a mesophilic, gram-positive, and cellulolytic bacterium (Sleat et al. 1984). Whereas *C. thermocellum* utilizes only cellulose, *C. cellulovorans* utilizes not only cellulose but also the components of hemicellulose such as xylan, fructose, galactose, and mannose (Tamaru et al. 2010). Based on whole-genome sequencing of *C. cellulovorans*, 57 cellulosomal protein-encoding genes and 168 secreted-type carbohydrase-encoding genes have been annotated (Tamaru et al. 2010; Matsui et al. 2013). Whereas *C. thermocellum* has a smaller number of such genes, *C. cellulovorans* has many genes encoding metabolic enzyme associated with interconversions between pentose and glucuronate, and metabolism of fructose, mannose, and galactose. The diversity of carbohydrases and metabolic enzymes in *C. cellulovorans* enables degradation and assimilation of various polysaccharides. In addition, *C. cellulovorans* optimizes carbohydrase production by modulating expression of metabolism-associated genes encoding cellulosomal and non-cellulosomal enzymes, depending on the availability of polysaccharides (Matsui et al. 2013; Aburaya et al. 2015; Esaka et al. 2015). Adapted production of carbohydrases and metabolic enzymes to extracellular polysaccharides enables the efficient degradation and assimilation of biomass. Although extensive research has been performed for assessing carbohydrase optimization and assimilation in media containing a single carbon source, not much research has been directed at how *C. cellulovorans* simultaneously assimilates polysaccharides in media containing more than two kinds of polysaccharides. In this study, we first investigated how *C. cellulovorans* assimilates oligosaccharides or polysaccharides in the media containing two kinds of carbon sources. We also discuss its characteristic system of polysaccharides incorporation and secretion of carbohydrases. This research provides the new insights for the utilization of *C. cellulovorans* to degrade lignocellulosic biomass.

## Material and methods

### Culture conditions

*Clostridium cellulovorans* 743B (ATCC35296) was grown anaerobically as described previously (Han et al. 2004), with the exception of carbon source in the media. As carbon sources, 2 % (w/v) glucose (Nacalai tesque, Kyoto, Japan), 2 % (w/v) cellobiose (Sigma, MO, USA), 2 % (w/v) microcrystalline cellulose (Merck, Darmstadt, Germany), 1 % (w/v) xylan (Sigma), 1 % (w/v) pectin (Sigma), 1 % (w/v) locust bean gum (LBG; galactomannan, Sigma), or 0.3 %  (w/v) phosphoric acid swollen cellulose (PASC) was used. PASC can be easily degraded compared to microcrystalline cellulose, because the cellulose in PASC is digested in smaller particles by the acid. PASC was prepared from microcrystalline cellulose as described previously (Zhang et al. 2006).

### Estimation of cell growth

Growth was measured by quantitation of intracellular ATP concentration by luciferase-based luminescence with a Lumitester PD-30 and LuciPac Pen (Kikkoman biochemifa, Tokyo, Japan) according to the manufacturer’s instruction. It is known that integrated intracellular ATP concentration correlates with cell growth (Miyake et al. 2016). Cell culture (100 μL) was incubated with 10 μL of ATP eliminating enzyme (Kikkoman) for 30 min at room temperature to remove extracellular ATP. Subsequently, cell growth was estimated by measuring ATP concentration of 100 μL of cell culture.

### Measurement of saccharide concentration in supernatant

Glucose and cellobiose concentrations in culture supernatants were measured by HPLC (Prominence; Shimadzu, Kyoto, Japan) equipped with an electrochemical detector (Coulochem III; thermo scientific, MA, USA). Supernatants were separated using a Sugar-D column (250 mm long, 4.6 mm inner diameter; Nacalai tesque) and the mobile phase was 80 % acetonitrile at a flow rate of 500 μL/min. The sample injection volume was 1 μL.

### Measurement of residual carbon source in the media

Residual carbon source in the culture supernatant and precipitation were measured. Cell culture (50 μL) were incubated with 930 μL of 50 mM citrate buffer (pH 5.0) and 20 μL of cellulase SS (Nagase Chemtex, Osaka, Japan) for 24 h at 50 °C. After degradation, reacting solution was centrifuged for 10 min at 13,000×*g*. Polysaccharide concentration was measured by GC–MS.

### GC–MS analysis

Degraded residual carbon sources (20 μL) and 60 μL of 2 mg/mL ribitol, as an internal standard were stored in a deep freezer (−80 °C) until frozen (30 min), and were subsequently lyophilized. Lyophilized sample was incubated with 20 mg/mL pyridine methoxyamine (Sigma) for 90 min at 30 °C. Silylation was performed using MSTFA (*N*-methyl-*N*-TMS-trifluoro-acetamide) (GL Sciences, Tokyo, Japan) for 30 min at 37 °C. We used a gas chromatograph GCMS-QP2010 ultra (Shimadzu) equipped with a capillary column (CP-SIL 8CB; 30 m, inner diameter. 0.25 mm; Agilent Technologies, CA, USA). The oven temperature was 80 °C at 0–2 min, increased to 200 °C at a rate of 100 °C per minute, then at a rate of 50 °C per minute to 330 °C, and was finally maintained at 330 °C for 5 min. The interface temperature was 250 °C, and the source temperature was 200 °C. Helium was the carrier gas and was set at a flow rate of 1.12 mL/min.

## Results

### Incorporation of saccharides

**Fig. 1:**
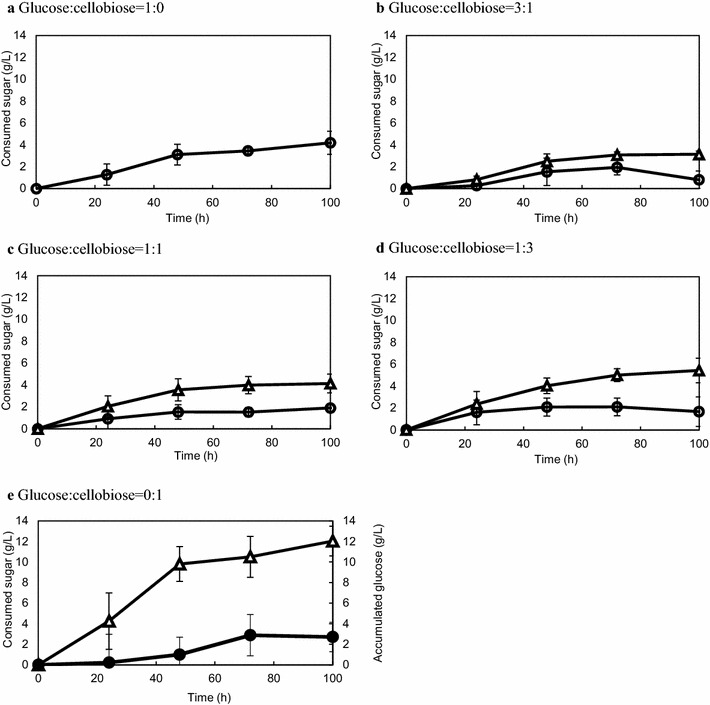
Saccharide assimilation by *C. cellulovorans*. *C. cellulovorans* was grown in media containing 20 g/L glucose (**a**), 20 g/L glucose-cellobiose mixture [glucose/cellobiose ratio of 3:1 (**b**), glucose/cellobiose ratio of 1:1 (**c**) and glucose/cellobiose ratio of 1:3 (**d**)], and 20 g/L of cellobiose (**e**). *Closed circles* glucose; *open triangles* cellobiose. All points were measured in triplicate. *Error bars* represent means ± SEs

First, we cultivated *C. cellulovorans* in media containing both glucose and cellobiose to investigate whether these sources were incorporated as monosaccharides or disaccharides. *C. cellulovorans* was grown in the media containing various ratios of glucose and cellobiose (glucose/cellobiose ratios were 1:0, 3:1, 1:1, 1:3, and 0:1 [w/w]). The consumption of cellobiose was greater than that of glucose, regardless of the mixture ratio (Fig. 1b–d). Furthermore, cellobiose was assimilated faster than glucose when comparing media containing only glucose and media containing only cellobiose (Fig. 1a, e). With media containing only cellobiose, glucose was produced and approximately 60 % of cellobiose was consumed after cultivation for 100 h (Fig. 1e). Approximately 20 % of consumed cellobiose was accumulated as glucose and was applied to growth (Fig. 1e). These results revealed that *C. cellulovorans* showed a preference for cellobiose compared to glucose.

### Cultivation in the media containing cellulose and hemicellulose

**Fig. 2:**
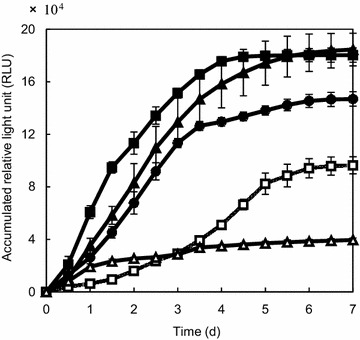
Comparison of cell growth in cellulose and cellulose-hemicellulose media. Growth curve of *C. cellulovorans* in the media containing 20 g/L of cellulose (*open square*), 20 g/L xylan (*open triangles*), 20 g/L of cellulose-xylan mixture (*closed circles*), 20 g/L of cellulose-pectin (*closed triangle*), and 20 g/L of cellulose-LBG (*closed squares*). All cellulose/hemicellulose ratio were 1:1 [w/w]. ATP (which correlates with cell growth) was represented as relative light units (RLU). All points were measured in triplicate. *Error bars* represent means ± SEs

Lignocellulosic biomass contains various polysaccharides such as cellulose and hemicellulose such as xylan, galactomannan (LBG), and pectin. We examined the polysaccharide assimilation profile of *C. cellulovorans* in the presence of both cellulose and hemicellulose. *C. cellulovorans* was cultivated in media containing three kinds of polysaccharides (cellulose-xylan, cellulose-LBG, and cellulose-pectin). *C. cellulovorans* grew better in media containing cellulose and xylan than media containing only xylan or cellulose (Fig. 2). Cultivation in media containing cellulose-pectin or cellulose-LBG also resulted in better growth than media containing only cellulose. The growth rate for the first 24 h (1 day) was nearly equivalent between cellulose-xylan and xylan media, which raises the possibility that *C. cellulovorans* could preferentially assimilate xylan until 24 h in the presence of both cellulose and xylan. In addition, the presence of LBG and pectin could have a similar effect as xylan, because growth in the media containing cellulose-LBG and cellulose-pectin showed the nearly same trend as that in cellulose-xylan media.

**Fig. 3:**
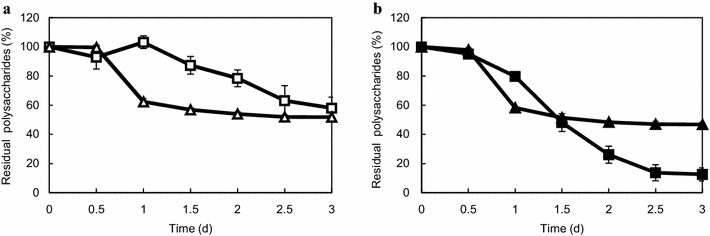
Comparison of residual polysaccharides in cellulose-xylan-containing and PASC-xylan-containing media. *C. cellulovorans* was cultivated in media containing 3 g/L of cellulose-xylan (**a**), (cellulose/xylan ratio of 1:1 [w/w]), and 3 g/L of PASC-xylan mixture (**b**), (PASC/xylan ratio of 1:1 [w/w]). The residual ratio of cellulose, xylan, and PASC were normalized to that at 0 h (set to 100 %). **a** *Open square* cellulose; *open triangles* xylan. **b** *Closed circle* PASC; *closed triangles* xylan. All points were measured in triplicate. *Error bars* represent means ± SEs

Next, the assimilation of each polysaccharide in the media containing cellulose and xylan was examined. Xylan was assimilated first, whereas cellulose was continuously assimilated (Fig. 3a). To elucidate whether slower assimilation of cellulose (compared to xylan) was caused by the difficulty of decomposing cellulose and easiness of decomposing xylan or carbohydrase production in *C. cellulovorans*, the assimilation profile for each polysaccharide in the media containing PASC (easier to degrade than cellulose) and xylan was compared to that of media containing xylan and cellulose. If PASC was assimilated faster than cellulose during the mixed cultivation with xylan, it implies that difficulty in decomposition of cellulose led to the slower incorporation of cellulose. In contrast, if the assimilation of PASC was comparable to that of cellulose, it implies that carbohydrase production is suitable for xylan assimilation. We observed that the assimilation of PASC was faster than cellulose (Fig. 3). This suggests that easiness of degradation determines the order of polysaccharide assimilation.

**Fig. 4:**
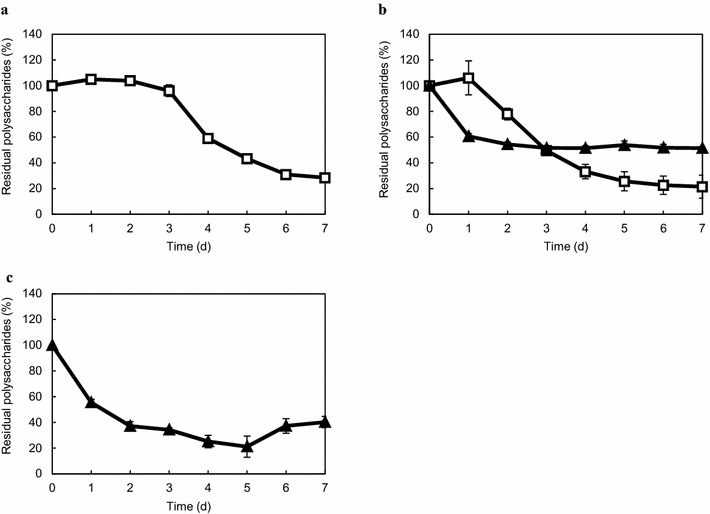
Comparison of residual polysaccharides in cellulose and cellulose-xylan, and xylan media. *C. cellulovorans* was cultivated in media containing 10 g/L of cellulose (**a**), 10 g/L of cellulose-xylan (cellulose/xylan ratio of 1:1 [w/w]) (**b**), and 10 g/L of xylan (**c**). The residual ratio of cellulose, xylan, and PASC were normalized to that of 0 h (set to 100 %). *Open square* cellulose; *closed triangles* xylan. All points were measured in triplicate. *Error bars* represent means ± SEs

Furthermore, faster growth through the presence of xylan in the media promoted cellulose utilization. *C. cellulovorans* assimilated cellulose after 24 h of cultivation in media containing cellulose and xylose, although the assimilation of cellulose started after 72 h (3 days) of cultivation in media containing only cellulose (Fig. 4). This suggests that the presence of xylan could increase the rate of cellulose assimilation.

## Discussion

Based on altering the cultivation media to contain various ratios of glucose and cellobiose, we observed that *C. cellulovorans* showed a preference for cellobiose. Cellobiose consumption was faster than that of glucose, and glucose was accumulated in the media containing cellobiose. Faster consumption of cellobiose than glucose was also observed in *C. thermocellum* (Ng and Zeikus 1982). In addition, *C. cellulovorans* has the ability to utilize xylan but not xylose (Sleat et al. 1984). These results interestingly suggest that *C. cellulovorans* incorporates oligosaccharides before degrading them to monosaccharides during polysaccharide utilization.

We cultivated *C. cellulovorans* in media containing three kinds of polysaccharides (cellulose-xylan, cellulose-pectin, and cellulose-LBG) to investigate the assimilation profile. *C. cellulovorans* grew better in media containing both cellulose and hemicellulose than that containing only cellulose. The results demonstrated that xylan was first assimilated, whereas cellulose was continuously utilized in cellulose-xylan media. Faster growth of *C. cellulovorans* in the presence of various carbon sources could be due to switching of target polysaccharides for assimilation from hemicellulose to cellulose. In the initial stage of growth, *C. cellulovorans* might consume hemicellulose which is easier to degrade before competitive cellulolytic bacteria can assimilate them. Non-cellulosomal and cellulosomal carbohydrases for hemicellulose and cellulose are secreted as it has grown, and therefore, cellulose can be degraded and assimilated. Thus, the presence of hemicellulose promoted cellulose utilization as well as growth, leading to efficient utilization of carbon sources in the media containing both of cellulose and hemicellulose. This characteristic is valuable for the utilization of lignocellulosic biomass, which contains not only cellulose but also hemicellulose.

Interestingly, *C. cellulovorans* incorporated little xylooligosaccharides in the media containing cellobiose, glucose, and xylooligosaccharides, suggesting that carbon catabolite repression must be caused by glucose and cellobiose (Additional file 1: Fig. S1). However, xylan was first assimilated in the media containing cellulose and xylan, (Fig. 4b). It is presumed that the assimilation pattern was different between oligosaccharides (Additional file 1: Fig. S1) and polysaccharides (Fig. 4b), because not only incorporation but also degradation is important for the assimilation ratio. Whereas *C. cellulovorans* could immediately incorporate oligosaccharides without degradation in the media containing oligosaccharide such as cellobiose and xylooligosaccharides, degradation is required, during growth in media containing polysaccharides such as cellulose and xylan. Xylan, which is easily degraded, was first assimilated despite carbon catabolite repression. To our knowledge, our results are the first to show the different response to oligosaccharides and polysaccharides, and further research is needed to investigate whether this phenomenon also occurs in other clostridia.

In this study, we clarified saccharide incorporation and assimilation in *C. cellulovorans*. This species incorporates oligosaccharides before degrading them to monosaccharides and grows effectively by switching the target substrates to be assimilated in the presence of various carbon sources, ranging from hemicellulose to cellulose. The presence of easily degraded carbon sources, especially hemicellulose, increases the growth and the assimilation rate of cellulose. These characteristics could make this species suitable for utilizing lignocellulosic biomass effectively, as this biomass contains various polysaccharides. Our findings provide new insight into the carbohydrate assimilation strategy of *C. cellulovorans* in the presence of various polysaccharides.

## Additional file

10.1186/s13568-016-0237-5 Oligosaccharide assimilation by *C. cellulovorans*. *C. cellulovorans* was grown in media containing 6 g/L glucose, 6 g/L cellobiose, and 6 g/L xylooligosaccharides mixture (a), 10 g/L glucose and 10 g/L xylooligosaccharides (b), 10 g/L cellobiose and 10 g/L xylooligosaccharides (c). Residual sugar amount was normalized to that of 0 h (set to 100%). Closed circles, glucose; open triangles, cellobiose; open squares, xylooligosaccharide. All points were measured in triplicate. Error bars represent means ± SEs.

## Abbreviations

ATCC: American type culture collection
HPLC: high performance liquid chromatography
