# Supplementary material for: Characteristic strategy of assimilation of various saccharides by Clostridium cellulovorans
Source: AMB Express. 2016 Sep 1;6(1):64. doi: 10.1186/s13568-016-0237-5 (PMC5009059; doi:10.1186/s13568-016-0237-5)
Supplement: Supplementary file 1 — 10.1186/s13568-016-0237-5 Oligosaccharide assimilation by C. cellulovorans. C. cellulovorans was grown in media containing 6 g/L glucose, 6 g/L cellobiose, and 6 g/L xylooligosaccharides mixture (a), 10 g/L glucose and 10 g/L xylooligosaccharides (b), 10 g/L cellobiose and 10 g/L xylooligosaccharides (c). Residual sugar amount was normalized to that of 0 h (set to 100%). Closed circles, glucose; open triangles, cellobiose; open squares, xylooligosaccharide. All points were measured in triplicate. Error bars represent means ± SEs. [file 13568_2016_237_MOESM1_ESM.pdf]

AMB Express

# Characteristic strategy of assimilation of various saccharides by *Clostridium cellulovorans*

Takako INAMORI[1,4], Shunsuke ABURAYA[1,2,5], Hironobu MORISAKA[1,3,6], Kouichi KURODA[1,7], Mitsuyoshi UEDA[1,3,§]

[1] Division of Applied Life Sciences, Graduate School of Agriculture, Kyoto University, Sakyo-ku, Kyoto, Japan; [2] Research Fellow of Japan Society for the Promotion of Science, Sakyo-ku, Kyoto, Japan; [3] Kyoto Integrated Science and Technology Bio-Analysis Center, Shimogyo-ku, Kyoto, Japan;

[4] inamori.takako.23c@st.kyoto-u.ac.jp; [5] aburaya.shunsuke.37r@st.kyoto-u.ac.jp;

[6] morisaka@kais.kyoto-u.ac.jp; [7] k_kuro@kais.kyoto-u.ac.jp

[§]Corresponding author

Mitsuyoshi UEDA

Tel:+81-75-753-6110; Fax:+81-75-753-6112

Email address: miueda@kais.kyoto-u.ac.jp

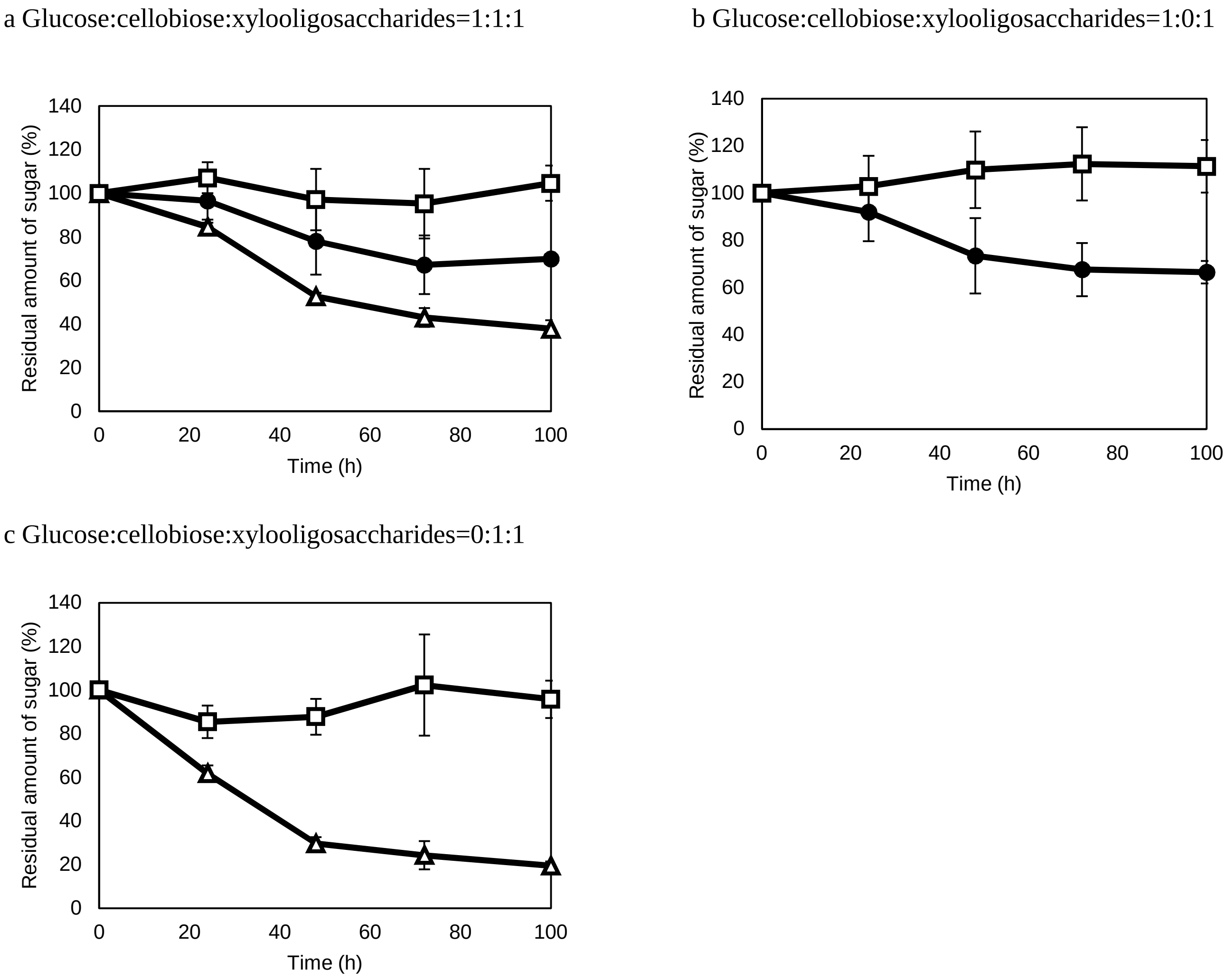


**Fig. S1 Oligosaccharide assimilation by *C. cellulovorans*.** *C. cellulovorans* was grown in media containing 6 g/L glucose, 6 g/L cellobiose, and 6 g/L xylooligosaccharides mixture (a), 10 g/L glucose and 10 g/L xylooligosaccharides (b), 10 g/L cellobiose and 10 g/L xylooligosaccharides (c). Residual sugar amount was normalized to that of 0 h (set to 100%). Closed circles, glucose; open triangles, cellobiose; open squares, xylooligosaccharide. All points were measured in triplicate. Error bars represent means ± SEs.
